# Evaluation of the Chemical Composition, Antioxidant and Antidiabetic Activity of *Rhaponticoides iconiensis* Flowers: Effects on Key Enzymes Linked to Type 2 Diabetes In Vitro, In Silico and on Alloxan-Induced Diabetic Rats In Vivo

**DOI:** 10.3390/antiox11112284

**Published:** 2022-11-18

**Authors:** Leyla Paşayeva, Hanifa Fatullayev, Ismail Celik, Gokhan Unal, Nuh Mehmet Bozkurt, Osman Tugay, Magda H. Abdellattif

**Affiliations:** 1 Department of Pharmacognosy, Faculty of Pharmacy, Erciyes University, Kayseri 38280, Turkey; 2 Department of Pharmaceutical Chemistry, Faculty of Pharmacy, Erciyes University, Kayseri 38280, Turkey; 3 Department of Pharmacology, Faculty of Pharmacy, Erciyes University, Kayseri 38280, Turkey; 4 Department of Pharmaceutical Botany, Faculty of Pharmacy, Selçuk University, Konya 42130, Turkey; 5 Department of Chemistry, College of Science, Taif University, Taif 21944, Saudi Arabia

**Keywords:** *Rhaponticoides iconiensis*, antioxidant, diabetes, α-amylase, α-glucosidase, LC-MS/MS, molecular docking, molecular dynamics

## Abstract

Diabetes mellitus (DM) is one of the globally worst killer diseases. In this study, the in vitro and in vivo antidiabetic activity and antioxidant capacity were determined and the phytochemical analyses were carried out on flower extract and sub-extracts of *Rhaponticoides iconiensis*. The in vitro antidiabetic activity was tested with α-amylase and α-glucosidase enzyme inhibition methods and an in vivo OGTT test in healthy and alloxan-induced rats. Although, the antioxidant activity was investigated with DPPH^●^, ABTS^●+^ and FRAP tests, the phytochemical composition analysis was carried out by LC-MS/MS. The highest α-glucosidase and α-amylase activity even from positive control acarbose were found in the ethyl acetate sub-extract of *R. iconiensis* (IC_50_ = 11.737 ± 0.823 µg/mL and 84.247 ± 0.721 µg/mL, respectively). This sub-extract also was active according to the results of in vivo tests. Moreover, the highest antioxidant activity on DPPH^●^ (IC_50_ = 0.126 ± 0.002 mg/mL), FRAP (at a concentration of 1 mg/mL equivalent to 3112.052 ± 2.023 mmol Fe^2+^) and ABTS^+●^ (at a concentration of 0.5 mg/mL equivalent to 0.608 ± 0.005 µM Trolox) tests. In addition, LC-MS/MS analyses of the active sub-extract revealed mainly the presence of patuletin, patuletin 3,7-diglucoside, naringin and 3,4-dicaffeoylquinic acid detected in the active sub-extract. In silico molecular docking and dynamics simulations studies were performed on these compounds with α-amylase and α-glucosidase enzymes for protein–ligand interactions and stability.

## 1. Introduction

Diabetes mellitus (DM) is one of the globally worst killer diseases. The International Diabetes Federation (IDF) has reported that approximately 552 million people will suffer from diabetes in 2030 [1]. Diabetes mellitus is a disease that occurs because of insulin deficiency. Impaired insulin effectiveness leads to elevated blood glucose levels, which cause oxidative stress (OS). Thus, by consuming antioxidants and controlling carbohydrate intake through inhibiting primary carbohydrate metabolizing enzymes, α-glucosidase and α-amylase, this disease can be prevented [2]. There is a wide range of synthetic antioxidative agents (such as butylated hydroxyanisole (BHA) and butylated hydroxytoluene (BHT)) and α-glucosidase and α-amylase inhibitors, which have side effects, namely, diarrhea, heart failure and flatulence. However, the usage of medicinal plants and natural products is increasing due to the fact of their wide range of pharmacological activity and minimum side effects.

It is well known that the Asteraceae family members’ use extends over the world for the treatment of various diseases. *Rhaponticoides* is a genera from this family that is separate from *Centaureae*. Based on previous reports, the species of the *Centaurea* genus are rich in bioactive secondary metabolites that are responsible for various biological activities; volatile components that are liable for antimicrobial effects; sesquiterpene lactones, flavonoids and lignans for cytotoxic and antitumor effects; and flavonoids and phenolic compounds for antidiabetic and antioxidant effects [3]. On the other hand, it is known that the species of this genus were used in traditional medicine from ancient times for their antifungal, antimicrobial, antidiabetic, anti-inflammatory and antiviral properties [4]. The genus *Rhaponticoides* is represented by 8 species in Turkey and 40 species around the world. The species found in Turkey are known by the local name “tülüşah”, and seven of them are endemic to Turkey [5,6].

There are some studies on *R. iconiensis* in the literature. In one study, the cytotoxic effect of the methanol extracts and sub-extracts of the leafy stem and flowers of *R. iconiensis* were investigated against the Colo 205, A549, HepG2, MCF7 and Beas-2b cell lines by the MTT method and the chemical compounds by LC-MS/MS. As a result, the methanol extract was found to be more cytotoxic to the Colo 205, HepG2, MCF-7 and Beas-2b cell lines, and the dichloromethane, ethyl acetate and *n*-hexane sub-extracts of the flowers and dichloromethane sub-extract of the stem were most cytotoxic against the A549, HepG2, MCF-7 and Colo 205 cell lines, respectively. According to the LC-MS/MS results, the methanol extracts mainly contained phenolic and organic acids and flavonoids [7].

In a study, the antioxidant properties and enzyme inhibition as well as the phenolic and flavonoid contents of the methanol (Soxhlet extraction and maceration) and water (infusion) extracts of *R. iconiensis* leaves, roots and flower heads were determined. According to the results, the methanol extracts of the leaves were found to be more active on the antioxidant tests and the methanol extracts of the roots were found to be most potent in the enzyme inhibitory tests, and hydroxybenzoic, hydroxycinnamic and acylquinic acids and flavones, flavonols, flavanones and anthocyanins were identified in the extracts [8].

In another study, the α-amylase and α-glucosidase inhibition activity, the antioxidant (with DPPH^●^, ABTS^●+^ and FRAP tests) antimicrobial properties (with the microdilution method), and the phytochemical composition by LC–MS/MS of the extract and sub-extracts from the leafy stem of *R. iconiensis* were evaluated. The ethyl acetate sub-extract was found to be the most active among the extracts, and the flavonoids, such as apigenin, naringin and patuletin derivatives, were determined to be the main compounds in the active sub-extract [9].

In this study, the methanol extract and n-hexane, dichloromethane, ethyl acetate, n-butanol and water sub-extracts from the flower of *R. iconiensis* were prepared, and the in vitro (α-amylase and α-glucosidase inhibiting activities) and in silico and in vivo antidiabetic activities, antioxidant (DPPH^●^, ABTS^●+^ and FRAP tests) effects and phytochemical substances (by LC-MS/MS) in the active sub-extract were investigated.

## 2. Materials and Methods

### 2.1. Plant Material and Reagents

After identifying the plant material, the aerial parts of *R. iconiensis* (Hub.-Mor.) M.V. Agab. & Greuter were collected from Konya, Seydişehir. The voucher specimens (Voucher No. 11.048) were stored in the Herbarium of Selçuk University.

All of the reagents and standards used in this study were purchased from Sigma-Aldrich (St. Louis, MO, USA), as were all of the enzymes, substrates, buffers and positive controls for the enzyme inhibition assays. The methanol and water were of LC grade and purchased from Merck Millipore (Billerica, MA, USA).

### 2.2. Extraction of the Plant Material

Before drying, the flowers of *R. iconiensis* were separated. The air-dried material (500 g) was powdered and extracted with methanol (3 L), by the maceration method, three times. At the end of the extraction, the obtained filtrates were pooled and evaporated in vacuo (37 °C). Then, the flower extract (TF) was dispersed with water and partitioned with *n*-hexane (TFH), dichloromethane (TFD), ethyl acetate (TFE) and n-butanol (TFB), sequentially. After the lyophilization process, the extracts were stored at −20 °C until the analysis time.

### 2.3. In Vitro Enzyme Inhibition Assays

In this study, the method of Paşayeva L. (2020) was used for the evaluation of the α-amylase and α-glucosidase inhibition activity [10]. For this assay, porcine pancreatic α-amylase was dissolved in phosphate buffer solution at a concentration of 4 U/mL. The enzyme solution (40 µL), 40 µL of extract/sub-extracts (40–2000 μg/mL in methanol) or control and sodium phosphate buffer solution were mixed in a plate. After preincubation at 25 °C, the starch solution (80 µL) was added and held at 25 °C for 10 min. Then, 80 µL of color reagent (dinitrosalicylic acid) was added and held at 85 °C in a water bath for 30 min. After the reaction was stopped, the absorbance of the samples was read at 540 nm in a microplate reader (Biotek Synergy HT, Winooski, VT, USA). In this study, the positive control was acarbose. The α-amylase inhibitory activity was calculated as per Equation (1):

(%) = [1 − (A_sample_/A_control_)] × 100 (1)

In this assay, 80 µL of α-glucosidase enzyme solution (2 U/mL) was prepared in phosphate buffer solution and then mixed with various concentrations of 80 µL of extracts/sub-extracts (3–120 μg/mL in methanol) on a plate. After preincubation for 10 min at 37 °C, 40 µL of 5 mM p-nitrophenyl-α-D-glucopyranoside was added and incubated at 37 °C for 20 min in a water bath (Memmert WNB45, Büchenbach, Germany). After adding 50 µL of 0.2 mM Na_2_CO_3_ solution, the absorbance was measured at 400 nm on a microplate reader (Biotek Synergy HT). In this test, acarbose was used as a positive control. The α-glucosidase inhibitory activity was calculated as described using the α-amylase method.

### 2.4. In Vivo Antidiabetic Activity

#### 2.4.1. Animals and Experimental Design

In this study, the active sub-extract TFE was investigated by in vivo antidiabetic tests. All experiments in this study were approved by Erciyes University Local Ethics Committee for Animal Experiments with the protocol number “20/152”. These experiments were carried out at Erciyes University, Experimental Research and Application Center (DEKAM). Male Wistar albino rats (12–15 weeks, 280–320 g) were used in the experiments. The experiments consisted of two distinct sub-experiments: Experiment 1 with healthy rats; Experiment 2 in alloxan-induced diabetic rats in this study. In Experiment 1, the rats were grouped as healthy control, TFE extract (300 mg/kg), TFE extract (600 mg/kg) and acarbose (5 mg/kg) (*n* = 5 each group). In Experiment 2, animals were grouped as saline, TFE extract (300 mg/kg), TFE extract (600 mg/kg) and acarbose (5 mg/kg) in treated alloxan-induced diabetic rats (*n* = 5 each group). We chose the 300 and 600 mg/kg doses of the TFE extract since they were found in the nontoxic doses of the preliminary toxicological tests (not presented here). The blood glucose levels in the oral glucose tolerance test (OGTT) were used to evaluate the antidiabetic effects of the extract and acarbose treatments in the healthy and alloxan-induced diabetic rats. The required amount of blood was taken from the lateral vein of the rat tail, and the glucose levels were evaluated by an automatic glucose meter (VivaChek Eco, Istanbul, Turkey).

#### 2.4.2. Alloxan-Induced Diabetes Model in the Rats

Alloxan monohydrate (Merck-Sigma Aldrich, Darmstadt, Germany) was dissolved in an appropriate volume of saline (final concentration: 100 mg/mL). The alloxan was injected daily (intraperitoneally) into the rats in a 100 mg/kg dose beyond three consecutive days. The fasted blood glucose levels were measured in 12 h starved rats, 72 h after the last injection. The rats which had higher blood glucose levels than 250 mg/dL were considered to have diabetes.

#### 2.4.3. Oral Glucose Tolerance Test (OGTT)

The OGTT was performed in both diabetic and nondiabetic healthy rats to measure postprandial blood glucose levels and to evaluate the antidiabetic effects of the extract and acarbose. The sub-extract (300 mg/kg), sub-extract (600 mg/kg) and acarbose (5 mg/kg) were orally administered 45 min before the OGTT. The glucose solution was prepared at a concentration of 2 g/mL and orally given to rats at a dose of 2 g/kg. The blood glucose levels were measured just before the administration (0 min), 30, 60, 90 and 120 min after the glucose solution [11].

### 2.5. The Total Phenolic (TPC) and Total Flavonoid (TFC) Contents

The TPC was performed using the method of Saeed et al. (2012) [12]. Fifty microliters of Folin–Ciocalteu (FC) reagent were mixed with 100 µL of ultrapure water and 50 µL extract/sub-extract (2, 1 and 0.5 mg/mL in methanol) in a plate. After 5 min, 100 µL of 7% Na_2_CO_3_ was added to start the reaction. After 90 min the absorbance was measured at 765 nm in a microplate reader (Biotek Synergy HT). The TPC was calculated as the gallic acid equivalents (GAE) per gram of extract.

The (TFC) of the extract and sub-extracts was estimated by the method of Marinova et al. (2015) [13]. According to this method, the sample solutions (50 µL) (2, 1 and 0.5 mg/mL in methanol), ultrapure water (200 µL) and NaNO_2_ (15 µL) were mixed in a plate. After 5 min, the AlCl_3_ (15 µL) and NaOH solution (100 µL) was added, and the absorbance was measured at 510 nm in a microplate reader (Biotek Synergy HT). The TFC was determined as a milligram of catechin equivalent. The TPC and TFC tests were carried out in triplicate.

### 2.6. In Vitro Antioxidant Activity Assays

#### 2.6.1. Assay for the DPPH Radical Scavenging Capacity

The 1,1-diphenyl-2-picrylhydrazyl radical (DPPH^●^) scavenging activity of the extract and sub-extracts were estimated according to the method of Gyamfi and Aniya (2002) [14]. In this assay, the different concentrations of the samples were obtained after adding a dilution of the stock solution (4 mg/mL in methanol). The concentrations were 0.025, 0.05, 0.1, 0.2, 0.4, 0.6, 0.8, 1 and 2 mg/mL. A volume of 20 μL of a sample concentration was mixed with 200 μL of DPPH^●^ solution in a plate. The mixture was left for 30 min at room temperature (25 °C), and then the absorbance was measured at 517 nm in a microplate reader (Biotek Synergy HT). In this study, butylated hydroxyanisole (BHA) was used as a positive control.

#### 2.6.2. Assay for the ABTS Radical Scavenging Capacity

The 2,2-azinobis (3-ethylbenzothiazoline-6-sulfonate) radical (ABTS^●+^) scavenging capacity was performed according to the method of Thaipong et al. (2006) [15]. One hundred and fifty microliters of the different sample concentrations (2, 1 and 0.5 mg/mL in methanol) were mixed with 2850 µL of the fresh ABTS^●+^ reagent (prepared by reacting 7,4 mM ABTS with 2.6 mM K_2_S_2_O_8_ in equal quantities, kept in the dark for 12 h). In this assay, BHA was chosen as a positive control, and the antioxidant capacity was determined as the Trolox equivalent. The absorbance was measured after 30 min in a microplate reader (Biotek Synergy HT) at 734 nm.

#### 2.6.3. Ferric Reducing Ability Power (FRAP)

To determine the antioxidant capacity of the extract and sub-extracts, the FRAP assay was estimated according to the method of Guo et al. (2003) [16]. For this, 40 µL of the extract/standard (2, 1 and 0.5 mg/mL in methanol) was mixed with 200 µL ultrapure water and 1800 µL of freshly prepared FRAP reagent. In this assay, the samples were incubated for 10 min at 37 °C, and the absorbance was recorded at 593 nm in a microplate reader (Biotek Synergy HT). The FRAP values were determined using FeSO_4_·7H_2_O as a standard ferric reducing activity. The results are expressed as mmol of Fe^2+^ equivalents per g of extract/fraction weight (mmol Fe^2+^/g).

### 2.7. Phytochemical Analyses by LC-MS/MS

The LC-MS/MS conditions were a Shimadzu LCMS-8040 (Kyoto, Japan) triple-quadrupole mass spectrometer (ESI) that was used to analyze the chemical compounds in the active sub-extract. For this reason, the TFE sub-extract was directly injected into the LC-MS/MS, and a Q3 scan (precursor ion) was performed. Each precursor ion was fragmented into product ions at different voltages (15-25-35-45), and the most common fragments were selected. The auto MRM function of the Shimadzu 8040 was used for the optimization of the selected parent ions and fragment ions.

### 2.8. In Silico Studies

#### 2.8.1. Molecular Docking

The molecular docking study was conducted using the Schrodinger 2022.2 version’s Maestro graphical user interface. It was selected from PDB for the target enzymes α-glucosidase (PDB ID: 5NN8 [14]) and α-amylase (PDB ID: 1OSE [15]). The missing residues in the 5NN8 structure were completed using the AlphaFold protein structure database [16]. Both of the target protein structures were prepared using the OPLS4 force fields [17] with the “Protein Preparation Wizard” with the default settings. The 3D structures of the naringin, patuletin, patuletin 3,7-diglucoside, 1,4-dicaffeoylquinic acid and acarbose were obtained from the PubChem database in the SDF file format and minimized with OPLS4 force fields using “LigPrep” and Epik at pH: 7 ± 2. The docking study was validated by redocking the cocrystal ligand of both enzymes with acarbose Glide SP [18]. Target α-glucosidase and α-amylase were docked with these compounds with Glide SP. The 2D and 3D protein–ligand interaction visualization and analysis were performed with BIOVIA Discovery Studio Visualizer v21.

#### 2.8.2. Molecular Dynamics Simulations

As in our previously reported studies [19,20], the MD simulation was performed using Gromacs 2021.2 version [21]. The necessary input files for the MD of the protein–ligand complexes obtained from the glide ligand docking study were created using the CHARMM-GUI server (https://charmm-gui.org/ accessed on 23 September 2022) [22,23,24]. The Triclinic water box was created with the TIP water model at 10 Å from the protein–ligand complex and neutralized by adding 0.15 M KCl. Five thousand steps minimization and 0.3 ns duration NVT/NPT ensemble equilibration stages were performed; 100 ns MD was simulated to 2 fs and 1000 frames were recorded. The topology files were created using CHARMM36m force fields [25,26]. The trajectory analyses were performed with gmx rms, rmsf and hbond scripts, and the graphs were made with QtGrace v0.2.6 tools. The MD animation videos were created with PyMOL Molecular Graphics System v2.5.2.

### 2.9. Statistical Analysis

The statistical analysis for the in vitro analyses was performed using GraphPad Prism Software (La Jolla, CA, USA). For the in vitro tests, statistically significant values were compared using one-way ANOVA with Tukey’s post hoc test, and *p*-values less than 0.05 were considered statistically significant.

Statistical analyses for in vivo experiments were performed using Graph Pad Prism software. A two-way analysis of variance (ANOVA) was conducted to evaluate the statistical differences in the blood glucose levels. Tukey’s post hoc test was used for the comparison of groups. The data are presented as the mean ± standard error of the mean (SEM), and *p* < 0.05 was accepted as a value of significance.

## 3. Results and Discussion

### 3.1. In Vitro Antidiabetic Activity

**Figure 1:**
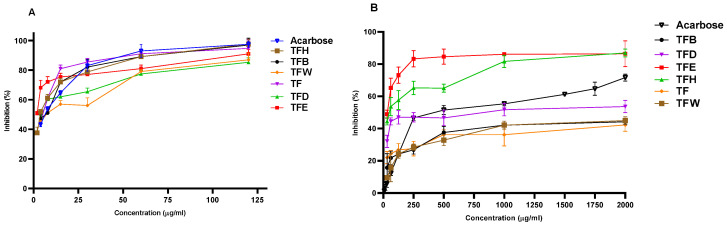
α-Glucosidase (**A**) and α-amylase (**B**) inhibitory activities of *R. iconiensis* flower extracts and sub-extracts.

An evaluation of the in vitro antidiabetic activities of the flower extracts and sub-extracts of *R. iconensis* was carried out with this study first. The results of α-glucosidase and α-amylase activities are described in Table 1 and Figure 1. According to the results of α-amylase’s inhibitory activity in the samples, the activity of TFE and TFD were higher (IC_50_ = 84.247 ± 0.721 and 93.912 ± 0.048 µg/mL, respectively) than acarbose as a positive control. On the other hand, other extracts and sub-extract showed moderate inhibitory activity. According to the results, the in vitro α-amylase inhibitory activities of the extracts obtained with different solvents were statistically significant between each other and the positive standard (*p* < 0.05).

In this study, the α-glucosidase inhibitory activity of TFM, TFH, TFB and TFE were found to be higher than the activity of acarbose (IC_50_ = 17.707 ± 0.569 µg/mL), and the TFE sub-extract was found to be the most active among the extracts (IC_50_ = 9.438 ± 0.599 µg/mL) and statistically different compared to acarbose.

### 3.2. Total Phenolic Content (TPC) and Total Flavonoid Content (TFC)

The TPC and TFC contents of the extracts and sub-extractions are described in Table 2. According to the results among the extracts, the TFE sub-extract contained higher total contents of phenolic compounds (292.333 ± 2.082 mg_GAE_/g_extract_) as well as total flavonoids (204.667 ± 1.528 mg_CA_/g_extract_) than the other extracts (*p* < 0.05).

### 3.3. In Vitro Antioxidant Activity Results

All results of the antioxidant properties of the extracts were given in Table 2. As a result of the DPPH^●^ antioxidant capacity, the TFE sub-extract was found to be more active than the other extracts, with 0.126 ± 0.002 mg/mL IC_50_ values. Furthermore, among the extracts, the TFH sub-extract showed the lowest antioxidant activity (IC_50_ = 0.756 ± 0.003 mg/mL). The DPPH radical scavenging activities of the extracts obtained with different solvents were statistically significant between each other and standard compound (*p* < 0.05).

The *R. iconiensis* flower extracts and the BHA concentrations were 0.5 mg/mL in order to determine the ABTS radical scavenging activity. According to the results, the highest ABTS activity was found in the TFE sub-extract than the others (0.608 ± 0.005 Trolox/g_extract_) (Table 2). According to the results, there was no statistically significant difference (*p* > 0.05) between the activities of the TFE sub-extract and the activity of the BHA. By the same TFE, the sub-extract was the most active (3112.052 ± 2.023 mmol Fe^2+^/g_extract_) in the FRAP assay, too (*p* < 0.05).

### 3.4. In Vivo Antidiabetic Activity

The OGTT was administered to the saline, sub-extract (300 mg/kg), sub-extract (600 mg/kg) and acarbose (5 mg/kg)-treated healthy rats. It was seen that there was no statistical difference in the fasting blood glucose levels among the groups in the healthy rats. The sub-extract (300 mg/kg), sub-extract (600 mg/kg) and acarbose (5 mg/kg) treatments provided significantly lower blood glucose levels than the saline-treated rats at the 30th min of the OGTT. The sub-extract (300 mg/kg) and sub-extract (600 mg/kg) caused higher blood glucose levels than the saline-treated rats 60 min after the glucose administration. There were no statistical differences in the blood glucose levels at the 90th and 120th min of the OGTT in the healthy rats (Table 3).

The effects of the sub-extract (300 mg/kg), sub-extract (600 mg/kg) and acarbose (5 mg/kg) were also investigated in the alloxan-induced diabetic rats with the OGTT. There was no statistical difference between the alloxan and treatment groups on the fasting blood glucose levels in the diabetic rats. The sub-extract (600 mg/kg) provided significantly lower blood glucose levels than the alloxan group at the 30th (*p* < 0.01), 60th (*p* < 0.01), 90th (*p* < 0.01) and 120th (*p* < 0.001) minutes, whereas the sub-extract (300 mg/kg) decreased (*p* < 0.05) the glucose levels at only the 120th minute of the OGTT. Moreover, the acarbose treatment significantly reduced the blood glucose levels at the 30th (*p* < 0.05), 60th (*p* < 0.001), 90th (*p* < 0.001) and 120th (*p* < 0.001) min of the OGTT compared to the alloxan group (Table 4).

### 3.5. LC-MS/MS Results

**Figure 2:**
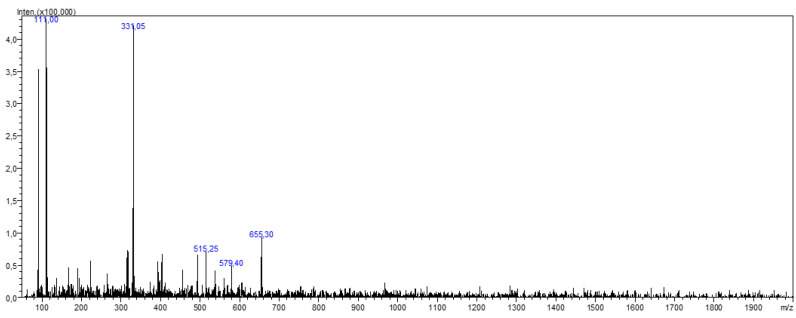
Mass spectra of the TFE sub-extract.

The identification of the compounds in the active TFE sub-extract was carried out by registered mass spectra fragmentation patterns, the NIST (National Institute of Standards and Technology) mass spectral database (version 2.3, Gaithersburg, MD, USA) and using literature data. According to the results, patuletin, patuletin 3,7-diglucoside [7], naringin [27] and 1,4-dicaffeoylquinic acid [8,28] were detected in the active sub-extract (Table 5 and Figure 2).

This is the first study to explore the antidiabetic and antioxidant activity as well as the phytochemical characteristics of the flower extract and sub-extracts of *R. iconiensis*. According to the literature data, there are some studies on the inhibition of the carbohydrate hydrolyzing enzymes and the antioxidant capacity of *R. iconiensis* species. In a study, it was described that the methanol extracts of the leaves and roots of *R. iconiensis* exhibited high α-glucosidase (2.48–3.08 mmol acarbose equivalent/g) and α-amylase (0.17–0.70 mmol acarbose equivalent/g) inhibition. The same study showed that the highest amount of phenolic (54.37 mg gallic acid equivalent/g) and flavonoids (80.75 mg rutin equivalent/g) were found in methanol extracts of *R. iconiensis* leaves. As described in this study, among the extracts, the Soxhlet extract and infusion from leaves showed the highest DPPH (131 and 132 mmol TE/g), ABTS radical scavenging activity (147 and 137 mmol TE/g) and FRAP assay (136 and 138 mmol TE/g) [8]. In another study, the α-amylase and α-glucosidase inhibition activity, the antioxidant, antimicrobial properties and the phytochemical composition of the extract and sub-extracts from the leafy stem of *R. iconiensis* were evaluated. As a result, the highest α-glucosidase and α-amylase activity were found in ethyl acetate sub-extract (TSE) of an *R. iconiensis* leafy stem extract (IC_50_ = 12.108 ± 0.582 µg/mL and 287.733 ± 0.325 µg/mL, respectively), and this extract was found to be more active on DPPH^●^ (IC_50_ = 0.249 ± 0.002 mg/mL), ABTS^●+^ (at a concentration of 0.5 mg/mL equivalent to 0.542 ± 0.002 µM Trolox) and FRAP (at a concentration of 1 mg/mL equivalent to 3642.005 ± 2.646 mmol Fe^2+^) tests. In addition, flavonoids, such as apigenin, naringin and patuletin derivatives, were determined to be the main compounds of the active TSE sub-extract [9].

Based on these results, it can be said that our results were higher than these results. It may be related to the extraction method. Knowing that high antidiabetic and antioxidant capacity is related to phenolic compounds, we obtained sub-extracts to increase the phenolics’ yield in this study. The highest TPC, TFC content, enzyme inhibition and antioxidant activities were found in the ethyl acetate sub-extract of *R. iconiensis*.

Although the in vivo antidiabetic activity of *R. iconiensis* was evaluated first in this study, our results showed that the ethyl acetate sub-extract of *R. iconiensis* reduced the blood glucose levels 30 min after the glucose solution in the healthy rats. Moreover, the extract, especially at a high dose, caused lower blood glucose levels beyond 120 min after the OGTT in the diabetic rats. Acarbose also decreased blood glucose levels during the OGTT in the diabetic rats and at the 30th min of the test in the healthy rats. These results demonstrate that the *R. iconiensis* sub-extract has potential against diabetes in the alloxan-induced diabetic rats.

The OGTT is a simple and widely used method for screening the potential effects of crude extracts or their chemical constituents on healthy rats’ glucose homeostasis. It is also beneficial for determining the antidiabetic potential of herbal extracts or drug candidates in diabetes-induced rodents [29]. There is no study on the antidiabetic effects of *R. iconiensis* or other members of the *Rhaponticoides* genus. There is only a limited number of studies on diabetes with close relative plants of *Rhaponticoides*. It has been demonstrated that *Centaurea alexanderina* leaf extract decreased the blood glucose levels in the OGTT in both diabetic and healthy rats [30]. In line with previous studies, our results first showed the antidiabetic and hypoglycemic effects of the ethyl acetate sub-extract of *R. iconiensis* in alloxan-induced diabetic and healthy rats, respectively. From a translational aspect, we suggest that the TFE sub-extract of *R. iconiensis* may hold promise, especially for diabetic people who have postprandial high glucose levels due to the fact of its proven α-amylase and α-glucosidase inhibitory effects, as shown by our study.

**Figure 3:**
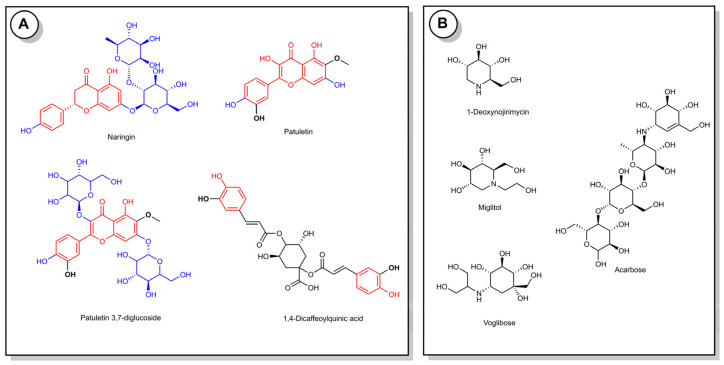
Chemical structures of identified compounds in the active sub-extract (**A**) and known α-glucosidase and α-amylase inhibitors (**B**).

Some investigations showed that the highly potent antidiabetic and antioxidant activity was related to the extracts’ high phenolic/flavonoid content. Furthermore, the phytochemical composition of the active sub-extract was also investigated, and we can say that the obtained results support the previous report [8]. Therefore, we can consider that the potent antidiabetic and antioxidant activity of the TFE extract may be explained by these compounds’ presence, especially flavonoids (Figure 3). The identified substances in the active sub-extract are already known for their antidiabetic properties and antioxidant activity [31,32,33,34]. Naringin is one of these compounds, exhibiting the most potent hypoglycemic activity via the inhibition of digestive enzymes, such as α-glucosidase and α-amylase with IC_50_ 0.55 and 8 µM, respectively, and was more active than acarbose [35]. Moreover, the antidiabetic effect of naringin in type 2 diabetic rats was also evaluated. As a result, it was demonstrated that naringin supplementation potentially ameliorated the elevated levels of glucose, glycosylated hemoglobin, AST, LDH and CK-MB and the lowered serum insulin level and hepatic and muscle glycogen content of insulin-resistant diabetic rats [31]. The treatments with naringin also enhanced the mRNA expression of insulin receptor β-subunit, GLUT4 and adiponectin in the adipose tissue of nicotinamide (NA)/streptozotocin (STZ)-induced type 2 diabetic rats [32,33]. These findings overlap with our results. Thus, the molecular docking studies also demonstrated the antidiabetic effect of these compounds. On the other hand, dicaffeoylquinic acids have been previously reported as constituents with strong antioxidant and hypoglycemic activity due the presence of functional groups (hydroxyl and caffeoyl groups) [36]. From the literature data, it was shown that the α-glucosidase and α-amylase inhibitory activities of dicaffeoylquinic acids at a concentration of 1 mM ranged from 65% to 62%. However, these compounds showed weak inhibitory activity against sucrase, isomaltase and porcine pancreatic α-amylase, ranging from 13% to 20% [37]. Although the effect of patuletin on diabetes has not been investigated, the antiproliferative, necrotic and apoptotic activity in tumor cell lines and anti-arthritic activity were investigated [38,39].

### 3.6. Molecular Docking and Dynamics Simulations

**Figure 4:**
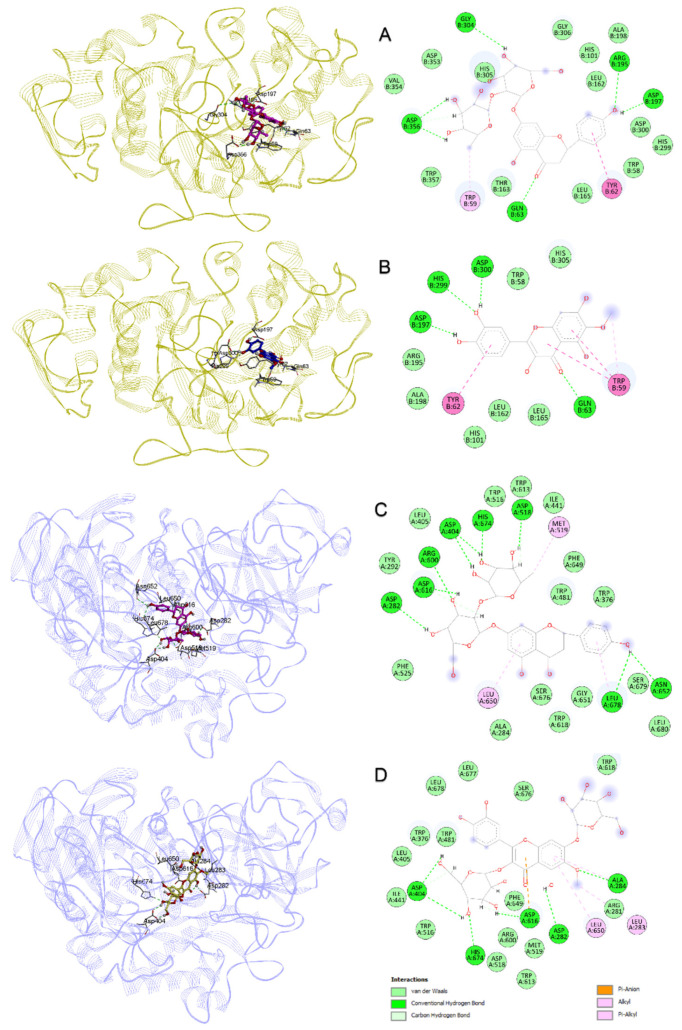
Binding poses and interaction diagram obtained from molecular docking: (**A**,**B**) compounds naringin and patuletin with α-amylase; (**C**,**D**) naringin and patuletin 3,7-diglucoside with α-glucosidase.

A molecular docking study was performed to explain the protein–ligand interactions of naringin, patuletin, patuletin 3,7-diglucoside and 1,4-dicaffeoylquinic acid, the compounds detected by the LC-MS analysis in the TFE sub-extract, with the target enzymes α-amylase and α-glucosidase [40]. First, the 1B2Y and 5NN8 IDs in the PDB were determined for the α-amylase and α-glucosidase 3D constructs, respectively. Redocking was performed with the cocrystal ligand acarbose Glide SP carried by each structure, and the maximum common structure RMSD value between the natural pose and docking pose was measured as 2.216 Å for 1B2Y and 1.429 Å for 5NN8 [41]. In general, values below 3 Å may indicate that the docking study is making a correct guess [22,42]. In Table 6, the compounds and docking scores of acarbose with α-amylase and α-glucosidase and protein–ligand interaction details are given. Naringin gave the lowest docking interaction energy, with both α-amylase and α-glucosidase. In Figure 4A,B, the binding poses and protein–ligand interaction diagrams of naringin and patuletin with α-amylase are provided. Naringin and patuletin formed H bonds with the residues of Gln63, Arg195 and Asp300, as in cocrystal acarbose, as shown in Figure 4C,D, between naringin and patuletin 3,7-diglucoside, and α-glucosidase resulted in H bond interactions with residues Ala284, His674, Asp616, Asp404 and Asp282.

**Figure 5:**
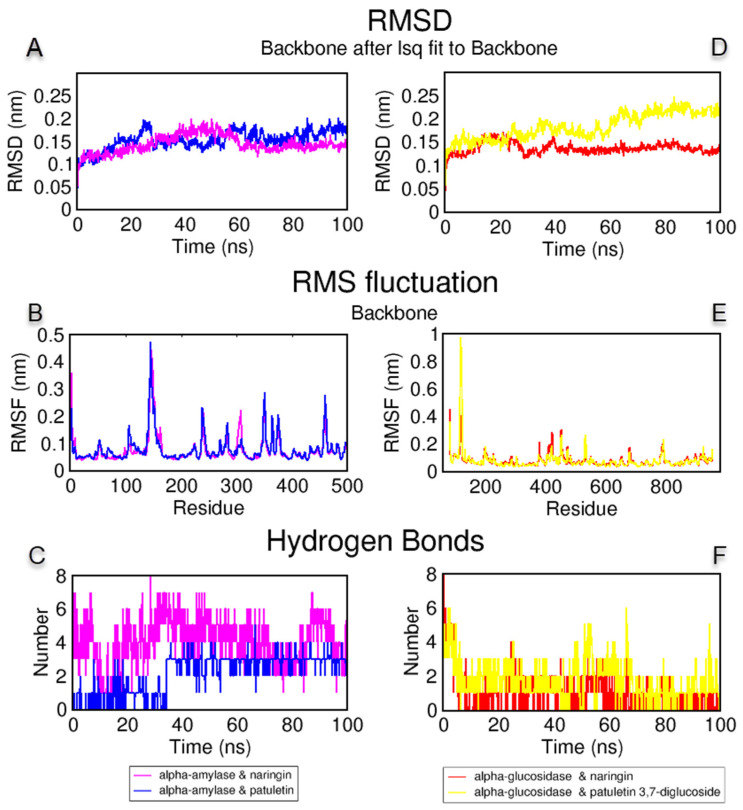
(**A**–**C**) The root mean square deviation (RMSD), root mean square fluctuation (RMSF) and protein–ligand hydrogen bond analysis and naringin and α-amylase and patuletin and α-amylase complexes; (**D**–**F**) RMSD, RMSF, H bonds plots of complexes naringin and α-glucosidase and patuletin 3,7-diglucoside and α-glucosidase for 100 ns.

An MD simulation was performed to examine the stability of the complexes obtained from the molecular docking study with naringin and patuletin with α-amylase; and naringin and patuletin 3,7-diglucoside with α-glucosidase. The root mean square deviation (RMSD), the root mean square fluctuation (RMSF) and the protein–ligand H bond analysis graphs obtained by the MD simulation trajectory analysis are given in Figure 5. The RMSD measurements were performed to analyze the shifts in the protein structure and mobility. As shown in Figure 5A,D, the complexes of naringin and patuletin with α-amylase were below 0.2 nm; the complex of naringin and α-glucosidase were at approximately 0.15 nm; and the complex of patuletin 3,7-diglucoside and α-glucosidase were at 60 ns and remained stable below 0.15 nm until then below 0.25 nm. The RMSF analyses were performed to analyze the flexibility per residue in the protein structure. As shown in Figure 5B, the naringin and α-amylase complex fluctuated less or close to each other in the patuletin and α-amylase complex, with the residues around Gln63, Arg195 and Asp300. The α-glucosidase complex of naringin and patuletin 3,7-diglucoside formed similar elasticities around the active site of amino acids, as shown in Figure 5D. The H bond is another type of analysis performed to demonstrate the protein–ligand stability. Over 200 ns, two to seven H bonds formed between naringin and α-amylase, and between one and four H bonds between patuletin and α-amylase, as shown in Figure 5C. Finally, H bond formations ranging from one to four were observed between naringin and α-glucosidase, and one to six between patuletin 3,7-diglucoside and α-glucosidase, as shown in Figure 5F. The MD animation videos of naringin and patuletin with α-amylase, in Supplementary Materials Videos S1 and S2, and naringin and patuletin 3,7-diglucoside with α-glucosidase, in Supplementary Materials Videos S3 and S4, demonstrate the protein–ligand interactions and stability provided in the Supplementary Materials as well as the interpretation and the experimental conclusions were drawn.

## 4. Conclusions

The present study concluded that the ethyl acetate sub-extract of *R. iconiensis* showed potential antidiabetic and antioxidant activity. The LC-MS/MS analyses revealed that the ethyl acetate sub-extract of *R. iconiensis* majorly comprised patuletin, patuletin 3,7-diglucoside, naringin and 3,4-dicaffeoylquinic acid. It can be deduced that in vivo antidiabetic activity of the sub-extract was mainly linked with the inhibition of carbohydrate metabolizing enzymes viz., α-amylase and α-glucosidase. The molecular docking studies hypothesized that the identified compounds were responsible for the enzyme inhibitory activity, especially naringin and patuletin in the α-amylase inhibition and naringin and patuletin-3,7-diglucoside in the α-glucosidase inhibition. The molecular dynamics simulations were performed to examine the stability of the docked compounds and enzymes. The MD simulations analyses revealed strong binding and stable complex compounds with enzymes Moreover, the limited studies on *Rhaponticoides* species and identified compounds combined with the activity evaluation will shed new light on the advanced studies.

## Figures and Tables

**Table 1:** α-Glucosidase and α-amylase IC_50_ values of the *R. iconiensis* flower extracts/sub-extracts.

Sample Names	α-glucosidase Assay IC_50_ (µg/mL)	α-amylase Assay IC_50_ (µg/mL)
TF	10.582 ± 0.470 ^a,d^	-
TFH	9.438 ± 0.599 ^b,a,d^	248.867 ± 0.547 ^a^
TFD	20.741 ± 0.089 ^c,g^	93.912 ± 0.048 ^b^
TFE	11.737 ± 0.823 ^d^	84.247 ± 0.721 ^c^
TFB	17.691 ± 0.587 ^e,g^	-
TFW	33.732 ± 0.940 ^f^	-
Acarbose	17.707 ± 0.569 ^g^	217.133 ± 0.001 ^d^

TF: methanol extract of *R. iconiensis* flower; TFH: *n*-hexane sub-extract of *R. iconiensis* flower; TFD: dichloromethane sub-extract of *R. iconiensis* flower; TFE: ethyl acetate sub-extract of *R. iconiensis* flower; TFB: *n*-butanol sub-extract of *R. iconiensis* flower; TFW: water sub-extract of *R. iconiensis* flower. Values are expressed as the mean ± SD (*n* = 3). Values are expressed as the mean ± SD (*n* = 3). Values with different letters (a–g) within each column are significantly different (*p* < 0.05).

**Table 2:** Total phenolic and flavonoid content and antioxidant activity results of *R. iconiensis* flower extracts and sub-extracts.

Samples	TPC (mg GAE/g_extract_)	TFC (mg CA/g_extract_)	FRAP (mmol Fe^2+^/g_extract_)	DPPH (IC_50_) (mg/mL)	ABTS (µM Trolox/g_extract_)
TF	70.002 ± 0.003 ^a^	23.500 ± 2.816 ^a^	26.000 ± 1.001 ^a^	0.331 ± 0.001 ^a^	0.284 ± 0.001 ^a^
TFH	26.100 ± 1.054 ^b,f^	16.647 ± 0.905 ^b^	2741.123 ± 1.152 ^b^	0.756 ± 0.003 ^b^	0.137 ± 0.002 ^b,f^
TFD	107.333 ± 2.065 ^c^	58.300 ± 1.539 ^c^	236.589 ± 0.045 ^c^	0.654 ± 0.004 ^c^	0.482 ± 0.003 ^c^
TFE	292.333 ± 2.082 ^d^	204.667 ± 1.528 ^d^	3112.052 ± 2.023 ^d^	0.126 ± 0.002 ^d^	0.608 ± 0.005 ^d,g^
TFB	161.001 ± 1.002 ^e^	46.633 ± 2.801 ^e,f^	1807.333 ± 2.517 ^e^	0.241 ± 0.001 ^e^	0.431 ± 0.001 ^e^
TFW	26.647 ± 0.905 ^f^	47.333 ± 2.003 ^f^	328.467 ± 0.945 ^f^	0.540 ± 0.005 ^f^	0.132 ± 0.002 ^f^
Trolox	-	-	7911.014 ± 1.468 ^g^	-	-
BHA	-	-	-	0.057 ± 0.001 ^g^	0.620 ± 0.001 ^g^

TF: methanol extract of *R. iconiensis* flower; TFH: *n*-hexane sub-extract of *R. iconiensis* flower; TFD: dichloromethane sub-extract of *R. iconiensis* flower TFE: ethyl acetate sub-extract of *R. iconiensis* flower; TFB: *n*-butanol sub-extract of *R. iconiensis* flower; TFW: water sub-extract of *R. iconiensis* flower. Values are expressed as the mean ± SD (*n* = 3). Values with different letters (a–g) within each column are significantly different (*p* < 0.05).

**Table 3:** Blood glucose levels of TFE (300 mg/kg), TFE (600 mg/kg) and acarbose (5 mg/kg)-treated healthy rats for fasting and OGTT.

Blood Glucose Levels (mg/dL)						
Treatment	Dose	0 min (Fasted)	30 min	60 min	90 min	120 min
Saline	-	119.3 ± 2.6	177.7 ± 4.3	108.7 ± 2.0	114.7 ± 2.9	113.3 ± 2.0
Sub-extract	300 mg/kg	117.5 ± 6.5	144.0 ± 12.4 *	148.0 ± 11.3 **	121.0 ± 4.5	118.8 ± 3.4
Sub-extract	600 mg/kg	106.0 ± 5.0	131.5 ± 5.0 ***	149.5 ± 5.5 **	128.0 ± 8.1	105.5 ± 4.0
Acarbose	5 mg/kg	100.8 ± 9.6	137.0 ± 4.6 **	112.0 ± 14.2	127.8 ± 11.4	110.0 ± 2.9

Data are presented as the mean ± SEM (*n* = 5). * *p* < 0.05; ** *p* < 0.01; *** *p* < 0.001 compared to the control group each time.

**Table 4:** Blood glucose levels of TFE (300 mg/kg), TFE (600 mg/kg) and acarbose (5 mg/kg)-treated rats in the alloxan-induced diabetic rats and healthy rats in the OGTT.

Blood Glucose Levels (mg/dL)						
Treatment	Dose	0 min (Fasted)	30 min	60 min	90 min	120 min
Alloxan	100 mg/kg	423.3 ± 14.5	529.7 ± 2.4	554.3 ± 14.3	561.3 ± 9.2	584.0 ± 8.3
Sub-extract	300 mg/kg	455.3 ± 10.1	480.0 ± 11.6	516.6 ± 44.1	513.3 ± 27.3	476.7 ± 12.0 *
Sub-extract	600 mg/kg	444.3 ± 6.3	448.4 ± 14.1 **	463.7 ± 23.4 **	480.0 ± 30.5 **	420.2 ± 17.4 ***
Acarbose	5 mg/kg	466.6 ± 8.8	461.0 ± 4.9 *	443.4 ± 3.5 ***	380.6 ± 18.3 ***	371.6 ± 4.4 ***

Data are presented as the mean ± SEM (*n* = 5). * *p* < 0.05; ** *p* < 0.01; *** *p* < 0.001 compared to the alloxan group each time.

**Table 5:** Mass spectral characteristics and the identity of the compounds in the TFE sub-extract.

[M-H]^−^ (*m*/*z*)	MS/MS (*m*/*z*)	Compounds	Reference
331	269, 183, 159, 151, 149, 117, 107, 83, 65	Patuletin	[7]
655	493, 331	Patuletin 3,7-diglucoside	[7]
579	271, 151	Naringin	[27]
515	353, 203, 191, 179, 173	1,4-dicaffeoylquinic acid	[8,28]

**Table 6:** Molecular docking interaction energies (kcal/mol) of the acarbose and compounds detected with the LC-MS analysis from the TFE sub-extract against the target enzymes, α-amylase and α-glucosidase.

		α-Amylase (PDB ID: 1B2Y)			α-Glucosidase (PDB ID: 5NN8)		
Compounds Name	PubChem ID	Docking Score	Protein–Ligand Interactions		Docking Score	Protein–Ligand Interactions	
			Hydrogen Bond	Hydrophobic		Hydrogen Bond	Hydrophobic
Naringin	442428	−7.635	Gln63 (2.15 Å), Arg195 (2.98 Å), Gly304 (2.83 Å), Asp356 (2.16 Å), Asp356 (1.77 Å and 2.56 Å), Asp197 (1.65 Å)	Trp59, Tyr62, Asp356	−6.930	Arg600 (2.10 Å), His674 (1.65 Å), Asp616 (1.61 Å), Asp282 (2.27 Å), Asp404 (2.11 Å and 1.84 Å), Asp51 (2.04 Å), Asn652 (2.82 Å), Leu678 (2.77 Å)	Leu650, Met519, Leu678
Patuletin	5281678	−7.360	Gln63 (2.09 Å), His299 (2.30 Å), Asp300 (1.72 Å), Asp197 (1.58 Å)	Trp59, Tyr62	−5.274	His674 (1.86 Å), Asp282 (1.76 Å), Asp616 (1.89 Å), Asp404 (2.04 Å)	Asp518, Asp616, Trp376, Phe649, Trp481, Phe525
Patuletin 3,7-diglucoside	44259785	−7.227	Arg195 (2.28 Å), Lys200 (2.02 Å), Lys200 (2.84 Å), Asp197 (1.54 Å), Asp300 (1.80 Å), Asp197 (1.86 Å), Gly306 (2.06 Å), His305 (2.00 Å)	Glu233, Asp300, Glu233, Glu240, Tyr151, Leu162, Ile235, Leu162	−5.511	Ala284 (2.32 Å), His674 (2.21 Å), Asp616 (1.72 Å), Asp404 (2.47 Å and 1.88 Å) and Asp282 (2.35 Å)	Asp404, Asp616, Leu283, Ala284, Leu650
1,4-Dicaffeoylquinic acid	12358846	−5.763	Trp59 (2.64 Å), Gln63 (2.15 Å), Thr163 (2.95 Å) Lys200 (1.97 Å), Asp356 (1.62 Å), Asp356 (1.93 Å)	Ile235	−4.832	Ala284 (2.64 Å), Trp481 (2.39 Å), Gly651 (2.72 Å), Asp282 (1.95 Å), Asp518 (1.70 Å), Ser676 (1.89 Å), Asp404 (2.59 Å)	Leu283, Ser676, Asp282, Asp616, Arg600, Asp518, Leu650, Ala284
Acarbose	445421	−8.480	Gln63 (1.99 and 2.79 Å), Arg195 (2.62 and 2.19 Å), Lys200 (2.71 Å and 2.06 Å), His299 (2.99 Å), Glu240 (2.44 Å), Gly306 (1.97 Å), His201 (1.93 Å), Glu233 (1.91 Å), Asp300 (1.94 and 2.52 Å), His305 (2.02 Å and 2.61 Å), Trp59 (1.83 Å)	Glu233, Asp300, Asp197, Gly306, His201, Thr163, Leu165	−6.130	Trp481 (3.04 Å), Arg600 (2.21 Å), His674 (2.23 Å), Asp282 (2.00 Å), Asp282 (2.04 Å), Asp616 (1.86 Å), Asp404 (1.89 Å and 2.02 Å)	Asp282, Asp518, Asp616, Asp404, Trp376, Trp481, Phe649

## Data Availability

The data is contained within the article and the Supplementary Materials.

## Supplementary Materials

The following supporting information can be downloaded at: https://www.mdpi.com/article/10.3390/antiox11112284/s1, Video S1: The molecular dynamics animations of 400 snapshots between 0 and 100 ns of the interaction between the compounds naringin and α-amylase; Video S2: The molecular dynamics animations of 400 snapshots between 0 and 100 ns of the interaction between the compounds patuletin and α-amylase; Video S3: The molecular dynamics animations of 400 snapshots between 0 and 100 ns of the interaction between the compounds naringin and α- glucosidase; Video S4: The molecular dynamics animations of 400 snapshots between 0 and 100 ns of the interaction between the compounds patuletin 3,7-diglucoside and α-glucosidase.
