# Supplementary material for: Evaluation of the Chemical Composition, Antioxidant and Antidiabetic Activity of Rhaponticoides iconiensis Flowers: Effects on Key Enzymes Linked to Type 2 Diabetes In Vitro, In Silico and on Alloxan-Induced Diabetic Rats In Vivo
Source: Antioxidants (Basel). 2022 Nov 18;11(11):2284. doi: 10.3390/antiox11112284 (PMC9686926; doi:10.3390/antiox11112284)
Supplement: Supplementary file 1 [file antioxidants-11-02284-s001.zip › antioxidants-1985866-supplementary.pptx]

## Slide 1
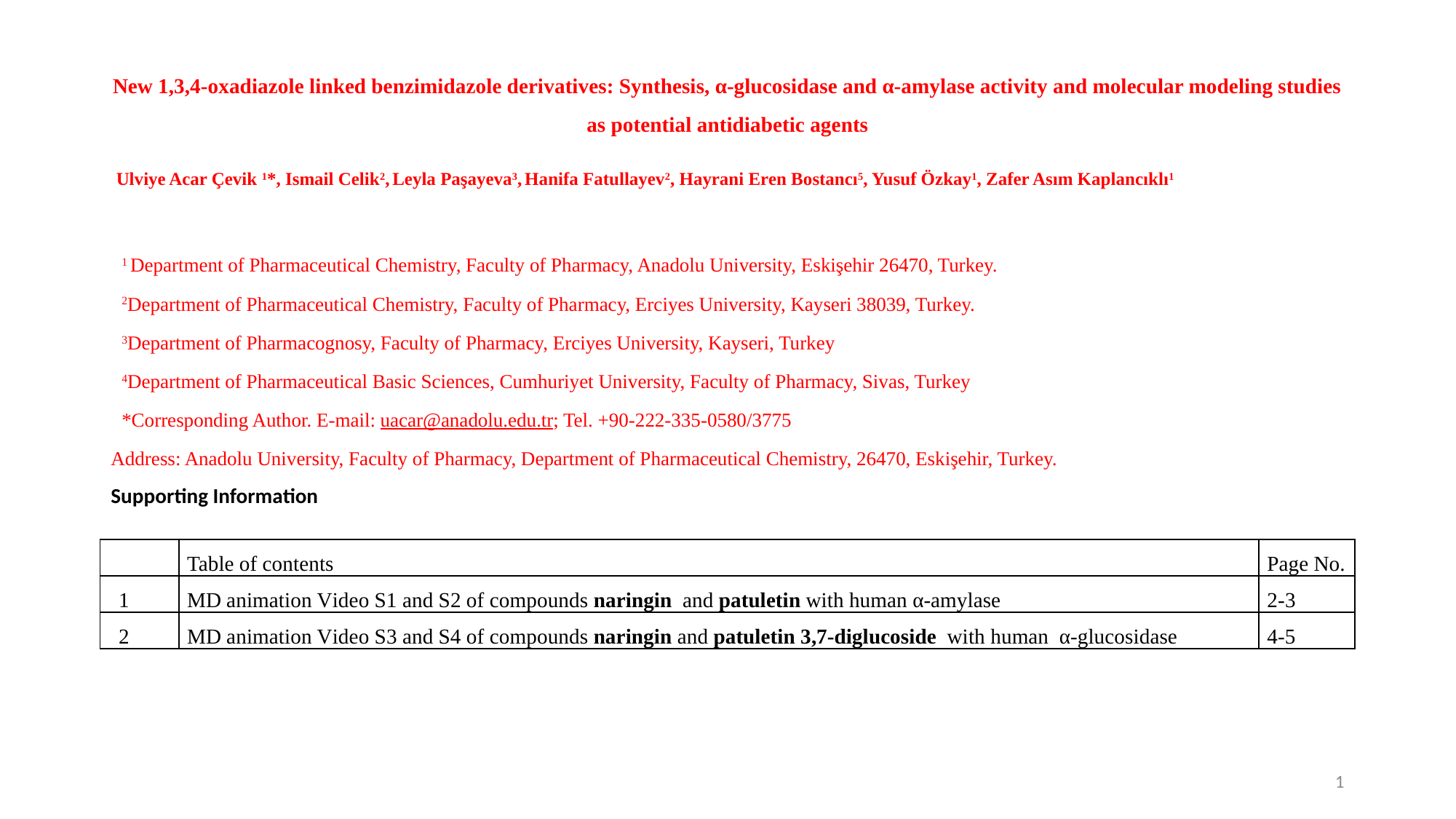

New 1,3,4-oxadiazole linked benzimidazole derivatives: Synthesis, α-glucosidase and α-amylase activity and molecular modeling studies as potential antidiabetic agents
 Ulviye Acar Çevik 1*, Ismail Celik2, Leyla Paşayeva3, Hanifa Fatullayev2, Hayrani Eren Bostancı5, Yusuf Özkay1, Zafer Asım Kaplancıklı1
1 Department of Pharmaceutical Chemistry, Faculty of Pharmacy, Anadolu University, Eskişehir 26470, Turkey.
2Department of Pharmaceutical Chemistry, Faculty of Pharmacy, Erciyes University, Kayseri 38039, Turkey.
3Department of Pharmacognosy, Faculty of Pharmacy, Erciyes University, Kayseri, Turkey
4Department of Pharmaceutical Basic Sciences, Cumhuriyet University, Faculty of Pharmacy, Sivas, Turkey
*Corresponding Author. E-mail: uacar@anadolu.edu.tr; Tel. +90-222-335-0580/3775
Address: Anadolu University, Faculty of Pharmacy, Department of Pharmaceutical Chemistry, 26470, Eskişehir, Turkey.
Supporting Information
| | Table of contents | Page No. |
| --- | --- | --- |
| 1 | MD animation Video S1 and S2 of compounds naringin and patuletin with human α-amylase | 2-3 |
| 2 | MD animation Video S3 and S4 of compounds naringin and patuletin 3,7-diglucoside with human α-glucosidase | 4-5 |
1

## Slide 2
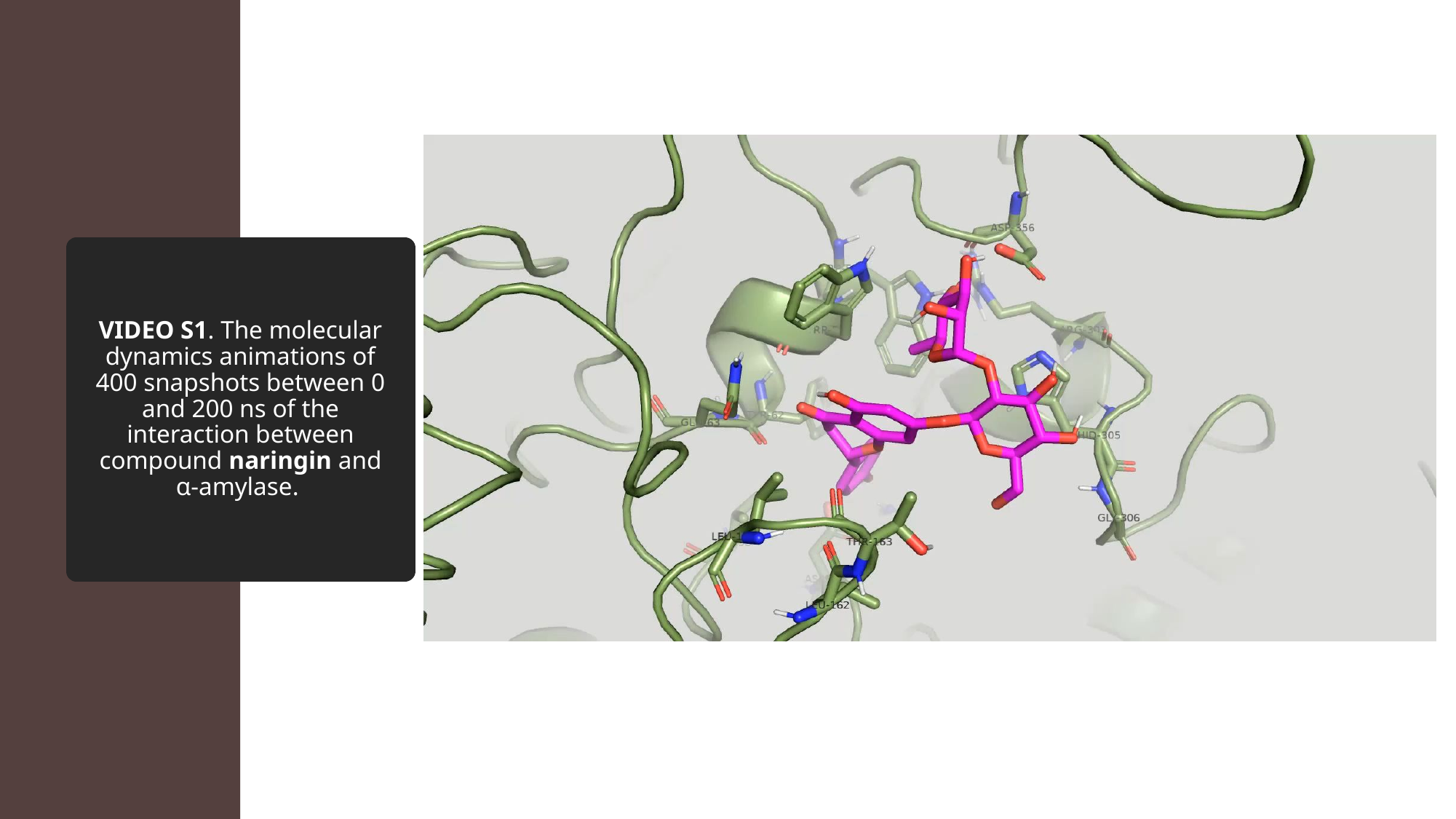

# VIDEO S1. The molecular dynamics animations of 400 snapshots between 0 and 200 ns of the interaction between compound naringin and α-amylase.
2

## Slide 3
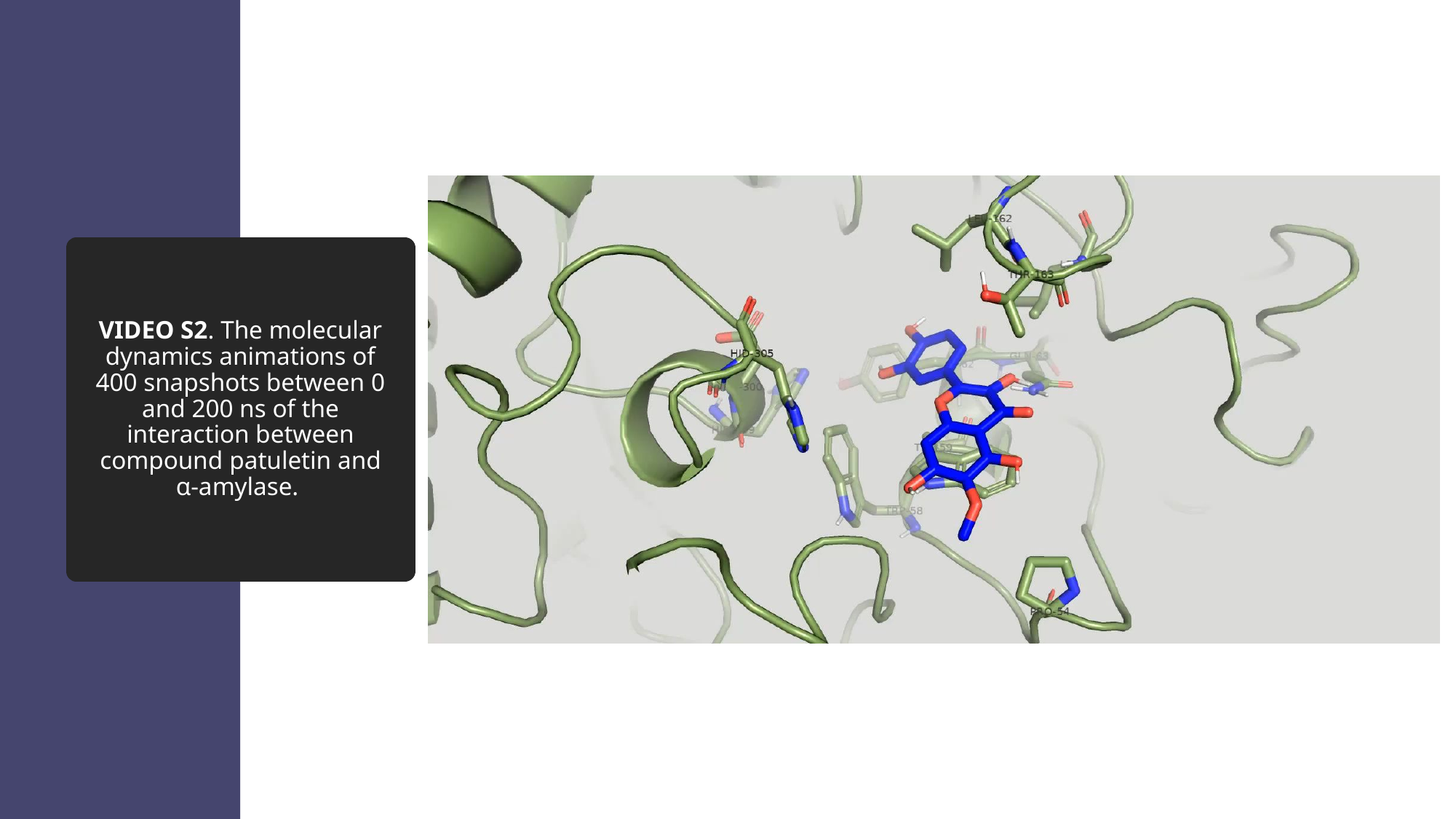

# VIDEO S2. The molecular dynamics animations of 400 snapshots between 0 and 200 ns of the interaction between compound patuletin and α-amylase.
3

## Slide 4
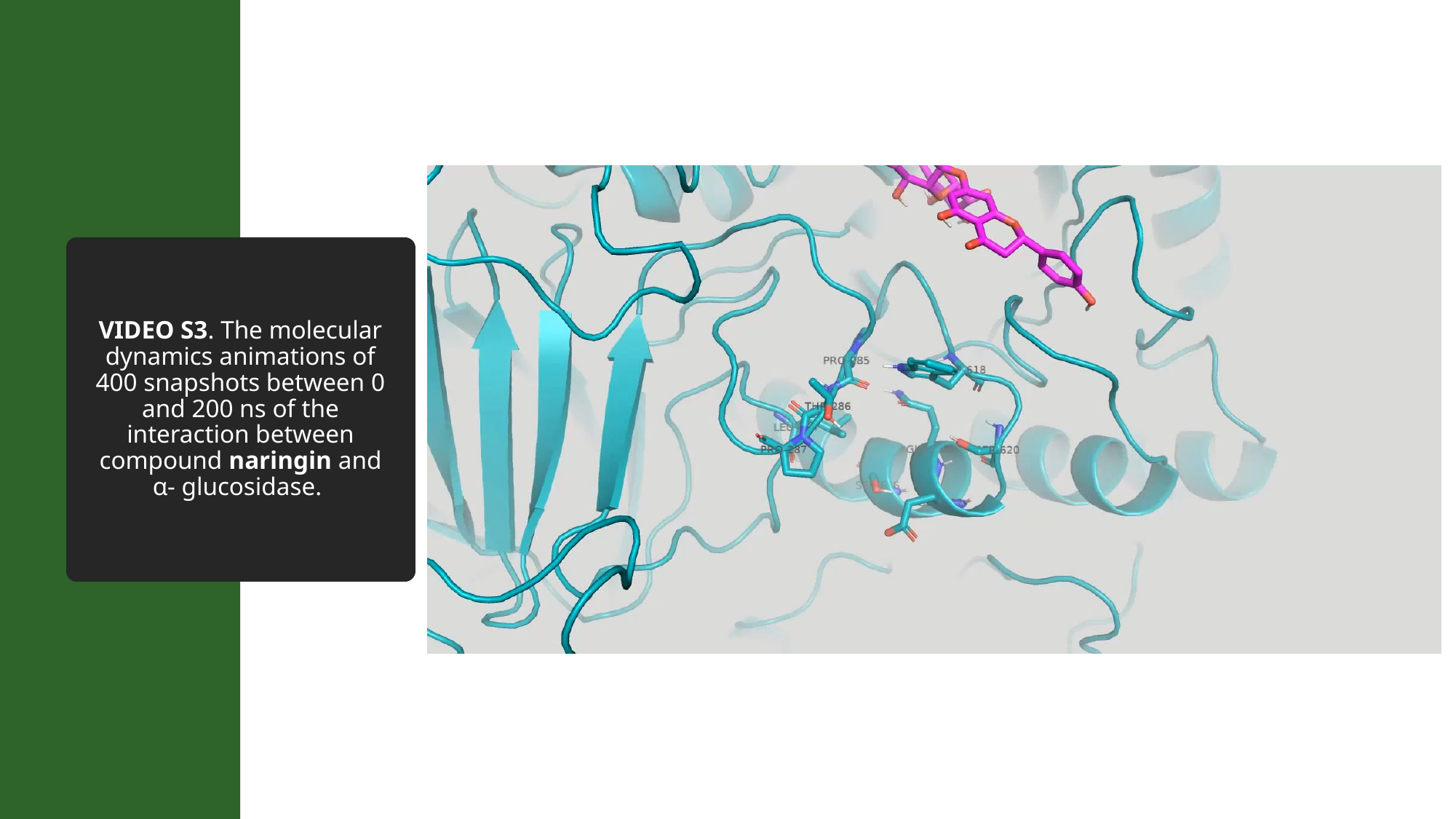

# VIDEO S3. The molecular dynamics animations of 400 snapshots between 0 and 200 ns of the interaction between compound naringin and α- glucosidase.
4

## Slide 5
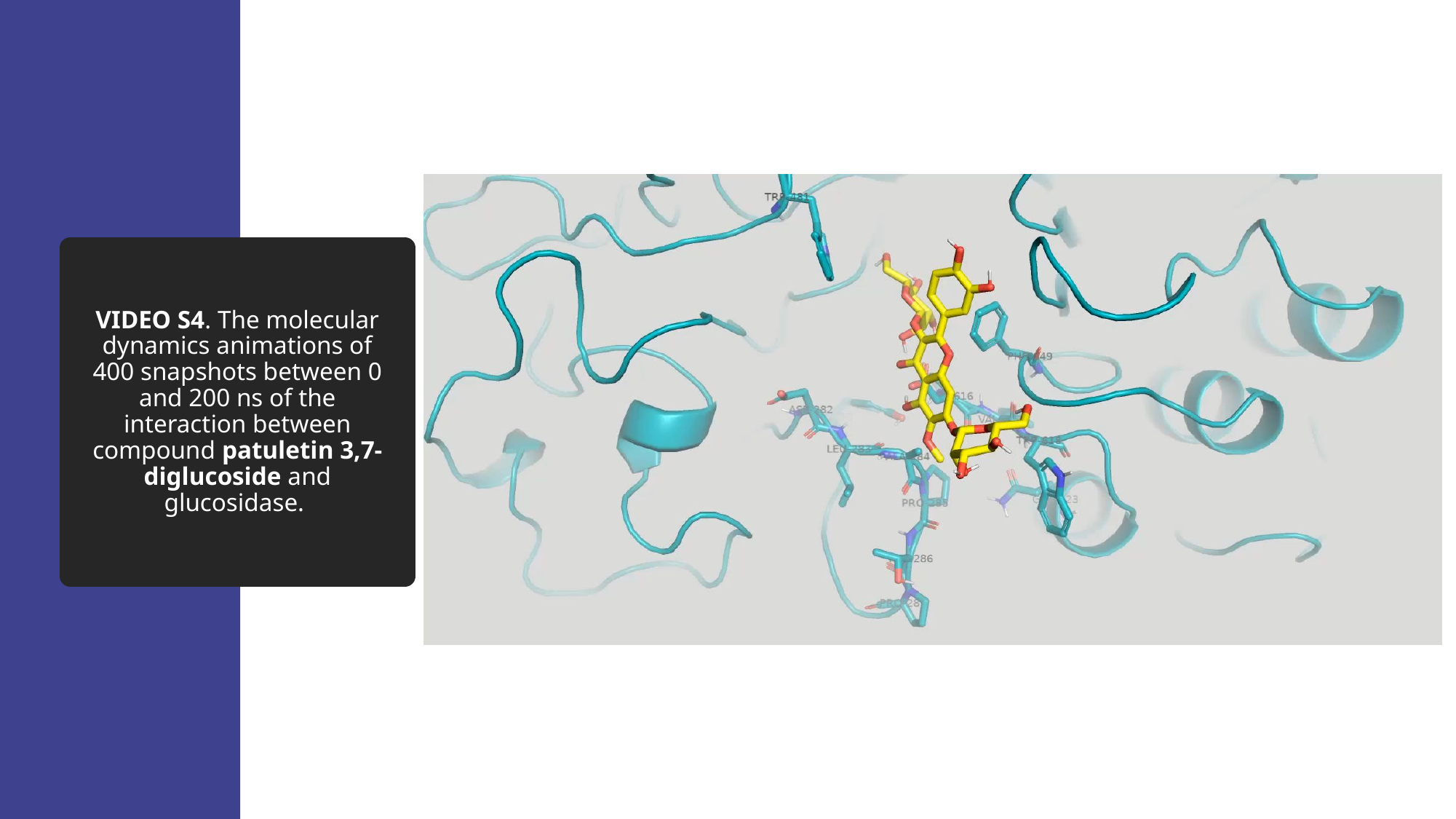

# VIDEO S4. The molecular dynamics animations of 400 snapshots between 0 and 200 ns of the interaction between compound patuletin 3,7-diglucoside and glucosidase.
5
